# Being a Parent of a Mentally Ill Child: A Study of Parental Stress and Mental Health

**DOI:** 10.1192/bjo.2026.11094

**Published:** 2026-06-30

**Authors:** Sheridan McWilliam, Nilamadhab Kar, Arif Khan, Sita Ratna

**Affiliations:** 1 Black Country Healthcare NHS Foundation Trust, Dudley, United Kingdom; 2 University of Wolverhampton, Wolverhampton, United Kingdom

## Abstract

**Aims:**

Children’s mental illnesses are negatively impacted by poor parental mental health. It is important to consider parents’ mental health for the welfare of their children. It is equally important to consider the impact of children’s mental health on the well-being and stress of their parents. The specific objectives of the study were to assess levels of psychological stress, anxiety, depression, and substance use as reported by the parents/caregivers. It was also ascertained if they had any psychiatric diagnoses and received intervention or support.

**Methods:**

This was a prospective, questionnaire-based survey of 146 parents attending a Child and Adolescent Mental Health Service (CAMHS) in the West Midlands. We used the Patient Health Questionnaire (PHQ-9) for depression and the Generalised Anxiety Disorder (GAD-7) questionnaire for anxiety. We assessed functional impairment through the Work and Social Adjustment Scale (WSAS).

**Results:**

The sample consisted of 25 fathers and 121 mothers, with the majority (90.9%) being of Caucasian ethnicity. Most of them were working, with 44.5% working full-time, 25.3% working part-time, and 25.3% being unemployed; 42.8% were receiving benefits. Proportions of parents who reported stress in the previous month “sometimes” was 23.4%, “often” 35.2%, and “almost always” 25.5%. Around one third (34.2%) reported having a psychiatric diagnosis; most of these were depression (22.6%). Based on PHQ score ≥10, 44.4% of parents reported symptoms suggestive of moderate to severe depression, and 39.5% had symptoms of moderate to severe anxiety (GAD-7 score ≥10). The anxiety and depression scores were positively correlated. Proportions of parents who reported smoking and using alcohol “often” or “most of the time” was 13.1% for each of these.

The majority (66.9%) had visited their GP for mental health-related issues or stress; a few had consulted a therapist or counsellor (47.9%), and 15.8% were seen by a psychiatrist. More than half (57.9%) of the parents were taking psychotropic medications; 9.0% used over-the-counter medications, and 6.9% used medication for sleep. Almost half of them had functional impairment, which included 28.7% having significant and 16.9% moderately severe or worse impairment.

**Conclusion:**

Considerable proportions of parents of mentally ill children had anxiety, depression, substance use, and functional impairment. Most of them sought help from professionals and received intervention. The results highlighted the extent of parental mental health concerns and the need for support. Besides improving awareness about this, options for screening of mental health problems in parents, and facilities for appropriate interventions are needed.

